# Unique bone microanatomy reveals ancestry of subterranean specializations in mammals

**DOI:** 10.1002/evl3.303

**Published:** 2022-11-11

**Authors:** Eli Amson, Torsten M. Scheyer, Quentin Martinez, Achim H. Schwermann, Daisuke Koyabu, Kai He, Reinhard Ziegler

**Affiliations:** ^1^ Staatliches Museum für Naturkunde Stuttgart DE‐70191 Stuttgart Germany; ^2^ Palaeontological Institute and Museum University of Zurich Zurich CH‐8006 Switzerland; ^3^ LWL‐Museum für Naturkunde Westfälisches Landesmuseum mit Planetarium DE‐48161 Münster Germany; ^4^ Research and Development Center for Precision Medicine University of Tsukuba Tsukuba 305‐8550 Japan; ^5^ Key Laboratory of Conservation and Application in Biodiversity of South China, School of Life Sciences Guangzhou University Guangzhou 510006 China

**Keywords:** Bone histology, compacted coarse cancellous bone, osteogenesis, phenotypic plasticity, subterranean mammals, Talpidae

## Abstract

Acquiring a subterranean lifestyle entails a substantial shift for many aspects of terrestrial vertebrates’ biology. Although this lifestyle is associated with multiple instances of convergent evolution, the relative success of some subterranean lineages largely remains unexplained. Here, we focus on the mammalian transitions to life underground, quantifying bone microanatomy through high‐resolution X‐ray tomography. The true moles stand out in this dataset. Examination of this family's bone histology reveals that the highly fossorial moles acquired a unique phenotype involving large amounts of compacted coarse cancellous bone. This phenotype exceeds the adaptive optimum seemingly shared by several other subterranean mammals and can be traced back to some of the first known members of the family. This remarkable microanatomy was acquired early in the history of the group and evolved faster than the gross morphology innovations of true moles’ forelimb. This echoes the pattern described for other lifestyle transitions, such as the acquisition of bone mass specializations in secondarily aquatic tetrapods. Highly plastic traits—such as those pertaining to bone structure—are hence involved in the early stages of different types of lifestyle transitions.

1 **Impact Summary**

Common selective pressures are often associated with the independent acquisition of similar traits in distantly related lineages. So what makes some lineages more evolutionarily successful than others? Here, we show that some groups of subterranean mammals have convergently evolved a specialized trait that involves the presence of large amounts of a rare bone tissue type, the compacted coarse cancellous bone. However, the true moles’ condition largely exceeds that of other digging mammals. This unique morphology can be detected in some of the earliest fossils of the family, suggesting that a bone microanatomy specialization might have had an important role in the evolution of true moles and predated the acquisitions reflected in their skeleton's external morphology. This echoes a similar pattern described in multiple lineages of vertebrates that acquired bone mass increase as part of their adaptation to an aquatic lifestyle. Specializations involving bone inner structure, which is highly adaptable throughout an individual's life, can hence be associated with different types of evolutionary transitions.

Lifestyle transitions drastically affect the evolution of many lineages. Such transitions, when examined at a large scale, are often associated with instances of convergent evolution (Ebel et al. 2020; Grossnickle et al. 2020; Martinez et al. 2020). Various clades of tetrapods that were ancestrally occupying aboveground niches have for instance independently acquired a subterranean lifestyle, entailing for these animals to spend most of their life underground (Nevo 1979; Sherratt et al. 2014; Ebel et al. 2020). Comparatively less studied, soil habitats are extremely biodiverse, and a growing body of evidence emphasizes their importance including for aboveground ecosystems functioning (Bardgett and Van Der Putten 2014). For tetrapods, the acquisition of a subterranean lifestyle hence enabled to access such rich habitats. This lifestyle is, however, associated with stringent selective pressures that are reflected in many aspects of their biology, including metabolism regulation, sense organs, and digging strategies (Nevo 1979). Although a serpentiform (“snake‐like”) morphotype has been repetitively acquired in amphibians and squamates (Woltering 2012), several mammalian diggers feature a “mole‐like” Bauplan (i.e., cylindrical body, expanded digging apparatus, eye reduction, and increase of tactile sense). The selective pressures associated with this Bauplan are so universal that this case of convergent evolution extends to insects (with the mole crickets, Gryllotalpidae [McGhee 2011]).

This subterranean syndrome can be framed in terms of evolution toward an adaptive optimum that drove the repeated acquisition of convergent traits (Losos 2011). This potentially concerns 13 independent lineages across extant therian mammals (placentals + marsupials; Fig. 1), which span about 160 million years (Ma) for the most distant taxa (Amson and Bibi 2021). These include, for instance, the Australian marsupial moles (*Notoryctes*), the African golden moles (Chrysochloridae), and the Holarctic true moles (Talpidae). To these specialized lineages with extant representatives should be added species for whom such adaptations are inferred from the fossil record, which already concerns the early evolution of mammaliaformes (Martin 2005), and even that of synapsids (e.g., Kammerer 2021). Playing a major role in locomotion in general and fossoriality in particular, the forelimb skeleton of subterranean mammals—both its gross morphology and inner structure—has been examined through comparative analyses (Kley and Kearney 2007; Hedrick et al. 2020; Sansalone et al. 2020; Pérez et al. 2021). Although the latter have identified features common to these specialized lineages, such as the large bony processes that improve the mechanical advantages of flexor muscles, little is known about the early stages of evolution of these specializations. Furthermore, some highly specialized digging styles are only practiced by single subterranean lineages, such as humeral rotation digging, with which drastic shifts in muscle attachment conformation are associated (Whidden 2000; Kley and Kearney 2007). Bone inner structure specializations have been argued to predate gross morphological ones in several transitions to the aquatic environment, where early stages of adaptations are better documented than for the subterranean clades (Madar 2007; Buffrénil et al. 2010). In the context of an aquatic adaptations, this pattern has been argued to be potentially linked to the highly plastic nature of bone inner structure (Amson et al. 2018), which can be adjusted throughout the life of an individual (Kivell 2016). Whether the same can be hypothesized for other lifestyle transitions, such as those involving subterranean clades, is also yet to be determined. More generally, a growing body of evidence points at the importance of phenotypic plasticity in great evolutionary transitions (Standen et al. 2014; Turko et al. 2019). However, finding other instances of macroevolutionary events involving phenotypic plasticity is difficult, as one should demonstrate that the specializations involved are indicative of a certain variability that has nevertheless been fixed, modifying the reaction norm of the trait in question (Pigliucci 2010).

**Figure 1:**
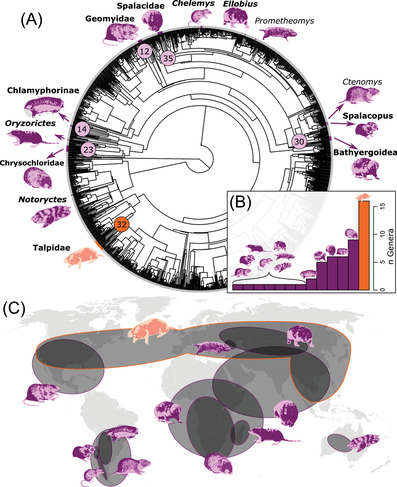
Mammalian acquisitions of the subterranean lifestyle. (A) Timetree of extant therian mammals (placentals + marsupials) genera with the 13 independent acquisitions of the subterranean lifestyle (those sampled here are in bold). Numbers at nodes are the diversification ages (millions of years) of the clades (no number for monogeneric clades). (B) Number of extant genera for each clade. (C) Approximate geographic distribution of each clade (based on IUCN 2021)

We examined the bone microanatomy of subterranean mammals, focusing in particular on the humeral structure. The humerus is very rich in anatomical features and has an exceptional fossilization potential, especially in some groups such as talpids (true moles and kin), making it ideal for comparative analyses (Sansalone et al. 2018). Talpids stood out in a dataset comprising 155 species representing most major groups of extant, terrestrial therian mammals. We further demonstrate that the bone histology is exceptional in talpids. Our study sheds light on a singular pattern of bone maturation that can be traced to some of the earliest fossils ascribed to the family.

## Methods

### STUDY DESIGN

A comparative analysis based on high‐resolution CT (computed tomography) data was combined to histological and developmental observations to (1) identify patterns of phenotypic evolution that concern the humeral bone microanatomy among subterranean mammals (and their close relatives) and (2) describe the bone histology and maturation in true moles (talpids), which stood out in the former analysis.

### SPECIMENS

The humerus of therian mammals with various lifestyles and body sizes were CT scanned at high resolution. The datasets of Amson (2021) and Amson and Bibi (2021) were extended with data acquired with micro‐CT scanners (Bruker SkyScan1272 and Nikon XTH 225 ST). Of particular importance for this work is the subterranean lifestyle, attributed based on Ellerman (1956). We follow the latter and classed the species as either subterranean or non‐subterranean (Table S5). The novel data (Table S5) relate to extant talpid species, among them the desmans, star‐nosed mole, and Japanese shrew mole. Even though the latter are not subterranean (Koyabu et al. 2017), they are grouped with other fossorial talpids (all species except shrew‐like moles, *Uropsilus* spp.) because of their fossorial habits and the fact that they are nested within an entirely subterranean clade. The extinct *Tegulariscaptor minor* was also added to the dataset. For a subset of species, skeletal elements of the rest of the forelimb, the hind limb, and vertebral column were also scanned. Specimens of extant species are wild‐caught museum specimens devoid of apparent disease. All specimens included in the quantitative analyses (see below) were skeletally mature (as indicated by epiphyseal fusion). Additional specimens at earlier ontogenetic stages were examined qualitatively. A bone histology dataset was built adding new thin sections of a humerus of *T. minor* (SMNS‐P‐47918; made following conventional methods [Meier et al. 2013]) to the data of Meier et al. (2013). Among the 13 lineages of extant mammals that independently evolved subterranean habits (Fig. 1), 11 could be included in the present study (specimens were excluded due to the presence of an epiphyseal line, which involved for two lineages to lack any representatives, see below). The fully aquatic taxa sampled by Amson and Bibi (2021) were excluded from the present analysis to avoid mixing their known bone mass specializations with the bone structure alterations described herein.

### PHYLOGENETIC TREE

The Maximum Clade Credibility DNA‐only node‐dated mammal phylogenetic tree of Upham et al. (2019) was modified according the content of our sample. The position of the extinct *Urotrichus giganteus* (age: 9.7 Ma, MN 10; Agustí et al. 2001) is incompatible with the divergence time of *Urotrichus* with *Dymecodon* (8.2 Ma; Upham et al. 2019). We therefore used the divergence time of the Timetree of life project (13.4 Ma; Kumar et al. 2017), and arbitrarily set the divergence between *Urotrichus* species halfway between the former divergence time and the age of *Urotrichus giganteus* (resulting branch length: 1.85 Ma; no uncertainty was included here because the measured traits closely resembled those of extant talpines).

The phylogenetic position of *Tegulariscaptor minor* (the species as a whole was assigned the maximum age of 33.7 Ma, Paleogene mammal zone MP 21 [Hopkins 2007; Ziegler 2012]) is not well resolved: the topology recovered by Sansalone et al. (2018) is not compatible with the timetree used here. We hence include uncertainty in setting different possible positions for *T. minor*: one as sister group of the non‐uropsiline talpids, and one as sister group of talpines (including, according to the topology used herein, Desmanini, *Condylura*, Talpini, Urotrichini, and *Scaptonyx*). For the first topology, the divergence times were set arbitrarily at 5% and 95% of the branch length (Trees 1A and 1B). For the second topology (Tree 2), the divergence age of talpines (as defined above; 29.4 Ma) had to be made older to accommodate the age of the fossil: this node's age was arbitrarily set 1 Ma older than the *T. minor*’s age.

### BONE MICROANATOMY

Microanatomical traits were quantified for the different compartments of the humerus, that is, the epiphyses and diaphysis. A volume of interest (VOI) was defined in the center of the head following Amson and Kilbourne (2019) (Fig. S1). The same approach was used to sample the capitulum (distal epiphysis). In the latter case, the VOI's center was defined by first locating the proximodistal middle of the capitulum; then, the mediolateral and anteroposterior position of the center is set by defining the center of a rectangle bound anteriorly and laterally by the edges of the capitulum, medially by the position of the maximal concavity of the anterior articular surface (capitulum/trochlea junction), and posteriorly at the maximal extension at the mediolateral middle defined by the previous steps. In case a VOI comprised the epiphyseal line (even partially), the specimen was excluded from the quantitative analyses. Because the trabeculae in these VOIs were found as compacted in most talpids, only two architectural parameters were relevant—the bone volume fraction (BV/TV, i.e., ratio between BV, the bone volume comprised in the VOI, and TV, total VOI volume) and the mean trabecular thickness (mean Tb.Th). These parameters were acquired with the software Fiji/ImageJ2 (Schindelin et al. 2012; Rueden et al. 2017) and the BoneJ2 plug‐in *Area/Volume fraction* and *Thickness* (Domander et al. 2021), after binarizing (Fiji plug‐in *Threshold*) of the sub‐stacks corresponding to the VOIs. Diaphyseal structure was assessed following Amson (2019). We examined in particular the mean global compactness, an approximation of the overall bone fraction of the diaphysis.

Similar to the epiphyseal VOIs, a VOI was also set for the mid‐lumbar vertebra (Amson and Bibi 2021) to compare the humeral structure to that of an axial skeleton element. Radial, ulnar, and femoral structures were also assessed, but only qualitatively, to include elements of the fore and hind limb long bones. All raw parameters are tabulated in Dataset S1.

### STATISTICAL ANALYSIS

All analyses were run with R version 4.0.5 (R Core Team 2021) taking into account the potentially confounding effects of body size and phylogenetic relatedness. Regressions and analyses of covariance (phyloANCOVAs) were performed with generalized least squares linear models assuming a within‐group correlation structure adjusted with the optimized Pagel's lambda value of the residuals (Revell 2010) (nlme package, *gls* function [Pinheiro et al. 2016]; APE package, *corPagel* function [Paradis et al. 2004]), and taking into account species variance heterogeneity (timetrees used are non‐ultrametric; *diag* and *vcv* functions [APE package] used to define the weights given as argument for the *gls* function). The Nagelkerke pseudo *R* ^2^ was computed with the piecewiseSEM package (Lefcheck 2016). Body mass was predicted both for extant and extinct species regressing extant species body mass (from the AnAge [Tacutu et al. 2013] and MOM version 1.4 [Smith et al. 2003] databases) against mean total cross‐sectional area of the humeral diaphysis (pseudo *R* ^2^ = 0.86; cross‐sectional area was not available for two specimens, for which we used the species body mass directly). The strength of the convergence among the 11 independent acquisitions of a subterranean lifestyle here sampled (see above) was assessed with a modified version of the package convevol (Stayton 2015; Zelditch et al. 2017). The latter was simply adapted for univariate analysis, enabling single variables to be provided as one of the function's arguments (Amson 2022). For that purpose, the timetree was modified so that each independent acquisition is represented by one tip. The mean trait value for each clade was used accordingly. The package phytools was used to produce phenograms (traitgrams) and mapping according to the different timetrees considered (functions *phenogram* and *contmap*, respectively [Revell 2012]). For the latter as well as the convevol analyses and violin plots (made with ggplot2 [Wickham 2016]), body size effect was accounted for using the residuals of regressions of the parameters of interest against body mass (the correlations were significant; Table S6).

## Results

### BONE MICROANATOMY AND HISTOLOGY

**Figure 2:**
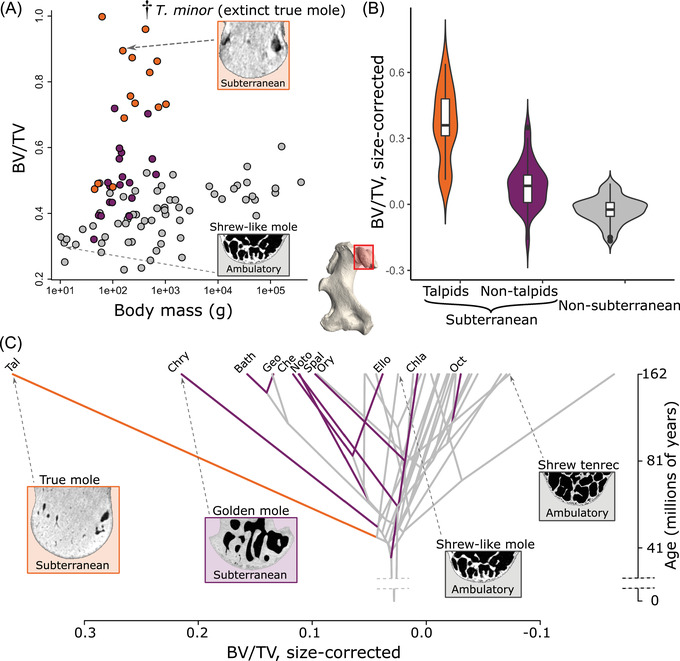
Evolution of humeral head bone fraction (BV/TV) in subterranean mammals. (A) BV/TV plotted against body mass. (B) Violin plots of the size‐corrected BV/TV among fossorial talpids (true moles), other subterranean species, and non‐subterranean mammals. (C) Phenogram depicting the size‐corrected BV/TV distribution among the clades that have independently acquired a subterranean lifestyle along with other mammals. The virtual cross sections are taken from the middle of the humeral head; bone is shown with gray values and intertrabecular space in black. The three‐dimensional rendering and virtual cross sections are not to scale. Bath = Bathyergoidea; Che = *Chelemys*; Chla = Chlamyphorinae; Chry = Chrysochloridae; Ello = *Ellobius*; Geo = Geomyidae; Noto = *Notoryctes*; Oct = *Spalacopus* (Octodontidae); Ory = *Oryzorictes*; Spal = Spalacidae; Tal = Talpidae

Subterranean mammals differ from the other sampled species in featuring significantly higher bone fraction in their humeral head (BV/TV; phyloANCOVA; *P* < 1 × 10^–7^ whatever the alternative topology in use; Fig. 2; Table S1). It is clear, however, that subterranean clades are not equally affected. The bone fraction values of most fossorial talpids exceed those measured in other clades (including when the effect of body size is accounted for). Seven of the 10 other subterranean clades display intermediate values (Fig. 2C). Despite this disparity among subterranean clades, their humeral head bone fraction is significantly convergent (C1 = 0.37; *P* < 0.038). Running the same analysis excluding talpids yields a clearer instance of convergence (C1 = 0.39; *P* < 0.042), showing that most talpids acquired a phenotype that exceeds the optimum toward which some other subterranean clades seemingly evolved. Moderately high to high bone fraction is found in all examined talpids except for the ambulatory shrew‐like moles, the sister group of the rest of the family (*Uropsilus* spp.; Fig. S2). Furthermore, exceptionally complete fossil humeri of *Tegulariscaptor minor* (early Oligocene, southern Germany)—the oldest and potentially most basal non‐uropsiline talpid for which humeral material is known—indicate that this distinctive phenotype is well expressed in the early history of the clade. A similar increase in bone fraction is also observed at the distal articulation (Fig. S3).

Examining the talpid humerus at the histological level, it is clear that this increase of bone fraction at the epiphyses (articular ends) is caused by the presence in large quantities of compacted coarse cancellous bone (CCCB; Fig. S4A). Not only the epiphyses, but the diaphysis (shaft) too is mostly made out of CCCB (Figs. S4B, S5). It is highly unusual to find the mid‐diaphyseal cortex (bone outer layer) made almost entirely out of CCCB, and to the best of our knowledge this was so far only documented in the aardvark (Legendre and Botha‐Brink 2018). The other long bones of the forelimb are also clearly sharing the same phenotype (Movie S1). The rest of the skeleton, however, greatly differs in its bone histology and microanatomy. Femoral thin sections at mid‐diaphysis are documenting the presence of mostly parallel‐fibered, avascular bone (Fig. S6), a more usual phenotype in small mammals (Kolb et al. 2015). Bone fraction measured in an axial skeleton element (mid‐lumbar vertebra) does not significantly differ among fossorial talpids, other subterranean clades, and the rest of the sample (Table S2).

The humeral diaphysis overall compactness is not affected by clear specializations in fossorial talpids as a whole. This conclusion has been reached based on mid‐diaphyseal cross sections (Meier et al. 2013), and is corroborated here with an assessment of the whole diaphysis (Fig. S7). Although there might be a trend for subterranean clades to feature a more compact diaphysis, the difference with non‐subterranean species is weak (phyloANCOVA, *P* > 0.17; C1 = 0.30, *P* > 0.25; Table S3). It is, however, clear that the amphibious desmans, the Russian desman in particular (*Desmana moschata*), display a particularly compact diaphysis. This is caused by a thickening of the cortex, with bone tissues representing about 85% of the total cross‐sectional area at mid‐diaphysis (and up to ∼96%; Figs. S8, S9).

### OSTEOGENESIS AND BONE MATURATION

**Figure 3:**
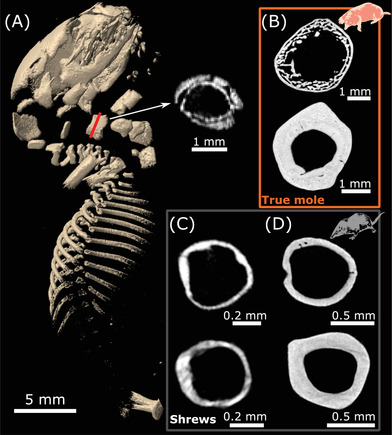
Talpid (true moles) bone microanatomy through ontogeny. (A) Three‐dimensional rendering and cross section of a middle stage fetus skeleton of *Scapanus orarius* (crown‐rump length = 27.5 mm; UMUT104497) with humeral cross section at growth center. Cross sections of subadult (top) and mature specimens (bottom): (B) *Condylura cristata* (top: SMNS‐Z‐MAM‐32943; bottom: NHMUK_ZE_1958.3.11.5), (C) *Suncus etruscus* (top: SMNS‐Z‐MAM‐49068; bottom: SMNS‐Z‐MAM‐49066), and (D) *Crocidura russula* (top: SMNS‐Z‐MAM‐43582; bottom: SMNS‐Z‐MAM‐43552). All cross sections were obtained through computed tomography and made at humeral mid‐diaphysis.

The ossification starts in talpids with a bone collar at mid‐diaphysis, as it is usual for long bones (early endochondral ossification; Fig. 3A). At the approximate equivalent of the mouse developmental stage E16.5, the humeral mid‐diaphyseal diameter represents but a fraction of that of the adult (Fig. 3B). Subadult specimens’ (90% or more of adult bone maximum length) μCT scans reveal a peculiar microanatomy in fossorial talpids, where the cortex is made of loose trabeculae with a roughly circumferential organization (Figs. 3B, S5, S10). This phenotype is somewhat reminiscent of the neonatal stage in the long bones usually observed in small mammals (Enlow 1962), with the important differences that early trabeculae in the latter are non‐lamellar and not as organized spatially. This pattern of bone maturation drastically differs from that of other small terrestrial mammals, where the cortex becomes compact at an early ontogenetic stage (Fig. 3B). The sub‐adult specimens sampled here evidence the fact that the final deposition of lamellar bone, filling up the inter‐trabecular spaces (and eventually yielding CCCB), occurs very late in the ontogeny of fossorial talpids. Indeed, the high bone fraction documented in the investigated VOIs is caused by an increase in trabecular thickness (phyloANCOVA; *P* < 1 × 10^–6^; Table S1).

## Discussion

### THE SINGULARITY OF TALPID SPECIALIZATIONS

Convergent evolution is ubiquitous across the tree of life, and it concerns every level of organization, from the species’ protein composition to their population structure (Anderson and Johnson 2009; Costa‐Paiva et al. 2018). Finding that multiple subterranean mammals have acquired a similar trait (increase of bone fraction in humeral head) is hence not surprising. Our results show, however, that talpids (true moles and their kin) represent a truly unique adaptation to extreme fossoriality: they acquired a bone microanatomy phenotype that exceeds the specializations shared by some of the other subterranean clades. Large amounts of lamellar bone eventually forming CCCB are found in both the humeral epiphyseal and diaphyseal compartments of all sampled talpids except for the ambulatory shrew‐like moles. Large amounts of CCCB at mid‐diaphysis were recently documented in a few other mammals: the aardvark (*Orycteropus afer*, for whom most of some long bone cortices are also mostly made out of it; Legendre and Botha‐Brink 2018), armadillos (Straehl et al. 2013; Heck et al. 2019), and mole rats (*Bathyergus suillus*; Montoya‐Sanhueza and Chinsamy 2017). The recurrent observation of this phenotype in fossorial species has already raised the question of its adaptive significance. Indeed, CCCB is regarded as a particularly compliant type of bone tissue, which could be beneficial for bones that are often under strong bending stresses (Legendre and Botha‐Brink 2018). The fact that the highly fossorial talpids feature the greatest proportion of CCCB—representing most of the epiphyseal and diaphyseal bone deposition—does corroborate this hypothesis. This question should be further investigated by examining bone histology in other systems under strenuous mechanical loading, be they appendicular skeletal elements or cranial ones (which are preponderantly involved in reptile and amphibian digging styles [Ebel et al. 2020]).

Our analysis also emphasizes that only six of the non‐talpid subterranean clades share an increase in the humeral head bone fraction, namely, the golden moles (Chrysochloridae), mole rats (Bathyergoidea), pocket gophers (Geomyidae), *Chelemys* (long‐clawed mouse), mole‐rats and kin (Spalacidae), the mole‐like tenrecs (*Oryzorictes*), and the marsupial moles (*Notoryctes*; Fig. 2C). This confirms that the subterranean lifestyle actually comprises disparate adaptations, as already pointed out (Nevo 1979; Kley and Kearney 2007; Hedrick et al. 2020; Amson and Bibi 2021). It is nevertheless remarkable that the forelimb of these six clades, which differ in many regards including the digging style they employ (chisel and/or scratch digging [Kley and Kearney 2007]) and their diet (insectivorous or herbivorous [Nevo 1979]), has partly evolved convergently.

### BONE MICROANATOMY VERSUS GROSS MORPHOLOGY

No humeral material is known for the earliest talpids, recovered from middle to late Eocene sediments (Hooker 2016). Our understanding of the early specializations affecting this skeletal element hence mostly relies on the exceptionally complete humeri ascribed to *Tegulariscaptor minor*, from the early Oligocene (Ziegler 2012; Sansalone et al. 2018). Its gross morphology specializations are viewed as intermediate (overall slenderness, short teres tubercle, and shallow brachialis fossa), differing substantially from the phenotype observed in extant fossorial talpids (Campbell 1939; Sánchez‐Villagra et al. 2004). We found that *T. minor* was fully expressing the bone microanatomy specializations otherwise observed in the latter (Figs. 2, 3, S5). This is consistent with an early acquisition of fossorial or subterranean habits. The ancestral lifestyle of non‐uropsiline talpids (all talpids but the ambulatory shrew‐like moles) is nevertheless still unclear, because semiaquatic/semifossorial (“*Condylura*‐like”) or semifossorial/ambulatory (“*Urotrichus*‐like”) habits cannot be ruled out. Furthermore, our results suggest that specializations pertaining to bone microanatomy evolved faster than gross morphology in talpids. Considering other lifestyle transitions preserved in the fossil record, it is noteworthy that a similar pattern occurred in the evolution of several lineages that adapted to the aquatic environment. This is the case in particular for sirenians, cetaceans, and thalassocnine sloths, which all acquired bone mass increase (BMI)—a pattern of bone structure alteration linked to buoyancy and trim adjustment (see also below)—early in their evolutionary history, before most other identifiable aquatic specializations (Madar 2007; Buffrénil et al. 2010; Cooper et al. 2012; Amson et al. 2014). Now extended to another type of lifestyle transition, the precocious acquisition of bone structure specializations hints at the widespread evolvability of bone microanatomy, possibly enabled by its high degree of plasticity (Lister 2021). The bone structure specializations in question, be it BMI or large amounts of CCCB, are not part of the phenotypic variation (reaction norm) normally observed in unspecialized taxa (and, in the case of talpids, not even in other subterranean clades). But increase of bone robusticity (not as severe as that associated with BMI) is known to be part of the plasticity of various skeletal elements (Lieberman 1996). Experimental data are required to test whether a similar pattern can be associated with CCCB deposition.

Bone structure plasticity can arguably also facilitate the acquisition of further specializations in an already highly specialized clade. In squamates, it has been argued that the subterranean lifestyle can be an “evolutionary dead‐end” (Ebel et al. 2020), likely because of the multiple and sometimes extreme modifications it is associated with. In talpids, however, we identified in the Russian desman a bone microanatomy phenotype consistent with the acquisition of an additional specialization to life in water. BMI was acquired independently in many semiaquatic or fully aquatic clades as part of the so‐called secondary adaptations of aquatic tetrapods (Buffrénil et al. 2010). The humerus mid‐diaphyseal compactness of the Russian desman approaches that of another amphibious mammal with a similar ecology, the platypus (*Ornithorhynchus anatinus*), for whom BMI has already been documented (Laurin et al. 2011). The Russian desman's body mass, however, is one order of magnitude smaller than that of the platypus, making it the smallest mammal with BMI. In the other amphibious talpids, the Pyrenean desman (*Galemys pyrenaicus*) and star‐nosed mole (*Condylura cristata*), the mean humeral diaphysis compactness is intermediate between those of the Russian desman and the shrew‐like moles. This is consistent with the fact that these other talpids are more incipiently adapted to water, which is also indicated by their gross morphology (e.g., whole tail not mediolaterally compressed as in the Russian desman [Howell 1930]) and less reduced olfactory turbinals (Martinez et al. 2020). Our results suggest that small‐bodied amphibious mammals, often overlooked in comparative analyses dealing with the influence of the aquatic environment, can be affected by alteration of bone structure reflecting their lifestyle. As a corollary, one can further conclude that the microanatomical specializations to fossoriality, which were acquired early in the evolutionary history of talpids, did not hamper the invasion of other habitats, as seen with the amphibious members of the clade.

### A SHORT LONG BONE

The Hox gene expression patterns have been found to differ widely in the talpid forelimb stylopodium when compared to that of the hind limb or the forelimb of the mouse (Bickelmann et al. 2017). The expression of *HoxD9*, in particular, is argued to be involved in the shortening of the humerus (demonstrated in the Iberian mole, *Talpa occidentalis* [Bickelmann et al. 2017]). We found copious amounts of coarse cancellous bone (CCCB) throughout the humerus in fossorial talpids. CCCB is usually absent at mid‐diaphysis because it is normally removed and replaced there during growth (Enlow 1962). In talpids, large amounts of CCCB are furthermore associated with a highly peculiar bone maturation process in the forelimb long bones, as it involves the maintenance of a cortex formed by lamellar trabeculae very late in the ontogeny. This phenotype seems to be decoupled from bone gross morphology specializations—bone shortening in particular—because talpids with relatively elongate bones (such as the star‐nosed mole or the desmans [Meier et al. 2013]) also display large amounts of CCCB throughout their diaphyses.

## Conclusions

We have demonstrated that true moles’ limb bone microstructure and histology fit into a broad pattern of convergent evolution associated with mammalian subterranean adaptations. True moles’ specializations, however, are hitherto unparalleled by any other subterranean clade. Although more data are necessary to clarify the exact function of these specializations, one can speculate that they have contributed to their evolutionary success: talpids represent one of the earliest therian acquisitions of the subterranean lifestyle, they are the most widespread geographically and the most diverse taxonomically (Fig. 1). Furthermore, the preservation of these microanatomical specializations in an early fossil of the group confirms that this kind of acquisition, which is arguably facilitated by the plasticity of bone structure, can be found at an early stage in different types of evolutionary lifestyle transition. This in turn lends support to the importance of phenotypic plasticity in macroevolutionary transitions.

## CONFLICT OF INTEREST

The authors declare no conflict of interest.

## AUTHOR CONTRIBUTIONS

EA designed research. EA, TMS, QM, AHS, DK, and KH acquired data. EA, TMS, QM, AHS, DK, KH, and RZ analyzed data and wrote the manuscript.

## DATA ARCHIVING

Computed tomography scan dataset was archived on MorphoSource (www.morphosource.org/projects/000478606). Custom R code is available on GitHub (https://doi.org/10.5281/zenodo.468447099).

## Supporting information

Legend for Movie S1

Figures S1 to S10
Tables S1 to S6

Legend for Dataset S1
