# Supplementary material for: Unique bone microanatomy reveals ancestry of subterranean specializations in mammals
Source: Evol Lett. 2022 Nov 11;6(6):552–61. doi: 10.1002/evl3.303 (PMC9783445; doi:10.1002/evl3.303)
Supplement: Supplementary file 2 — Figures S1 to S10 Tables S1 to S6 [file EVL3-6-552-s002.docx]

Supplementary Materials for

**Unique bone microanatomy reveals ancestry of subterranean specializations in mammals**

Eli Amson*, Torsten M. Scheyer, Quentin Martinez, Achim H. Schwermann, Daisuke Koyabu, Kai He, Reinhard Ziegler

*Corresponding author. Email: [eli.amson@smns-bw.de](mailto:eli.amson@smns-bw.de)

**This file includes:**

Figs. S1 to S10

Tables S1 to S6

**Other Supplementary Materials for this manuscript include the following:**

Movie S1

Data S1


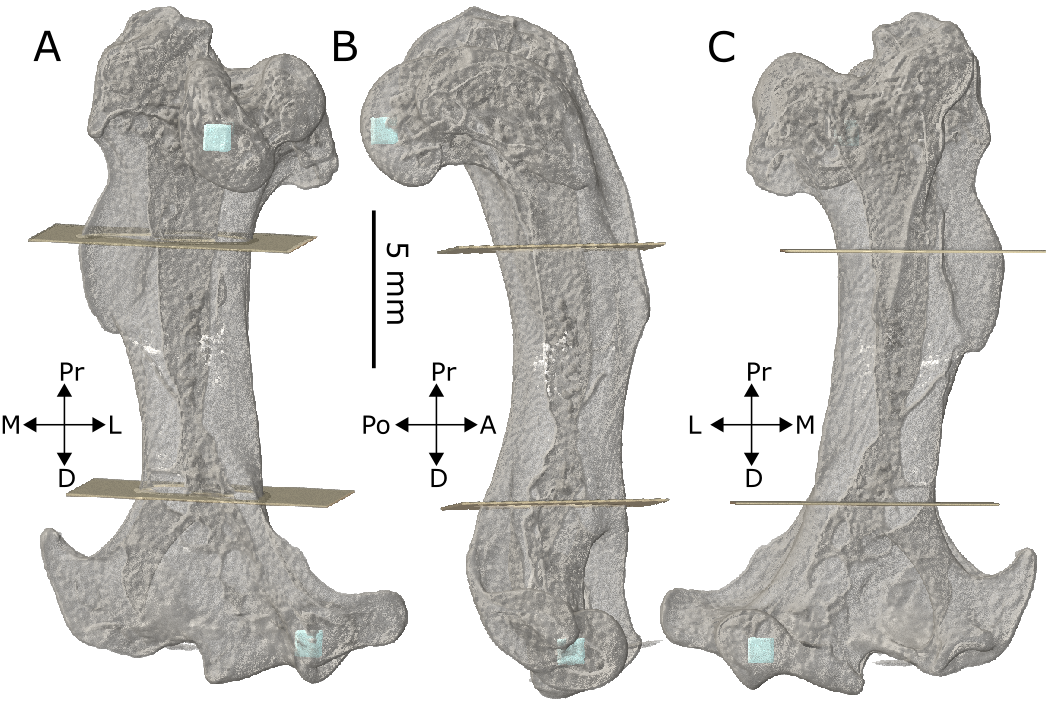


Fig. S1.

Location of the volumes of interest (VOIs, blue cubes) used to sample the trabecular properties of the humeral head (top, proximal) and capitulum (bottom, distal), as well as diaphyseal levels (orange planes) defining the first and last slices between which cross-sectional properties were acquired (see also Fig S9). Right humerus semi-transparent 3D rendering (*Desmana moschata*, SMNS-Z-MAM-160) seen in A, posterior, B, lateral, and C, anterior views. Abbreviations: A, anterior; D, distal; L, lateral; M, medial; Po, posterior; Pr, proximal.

**
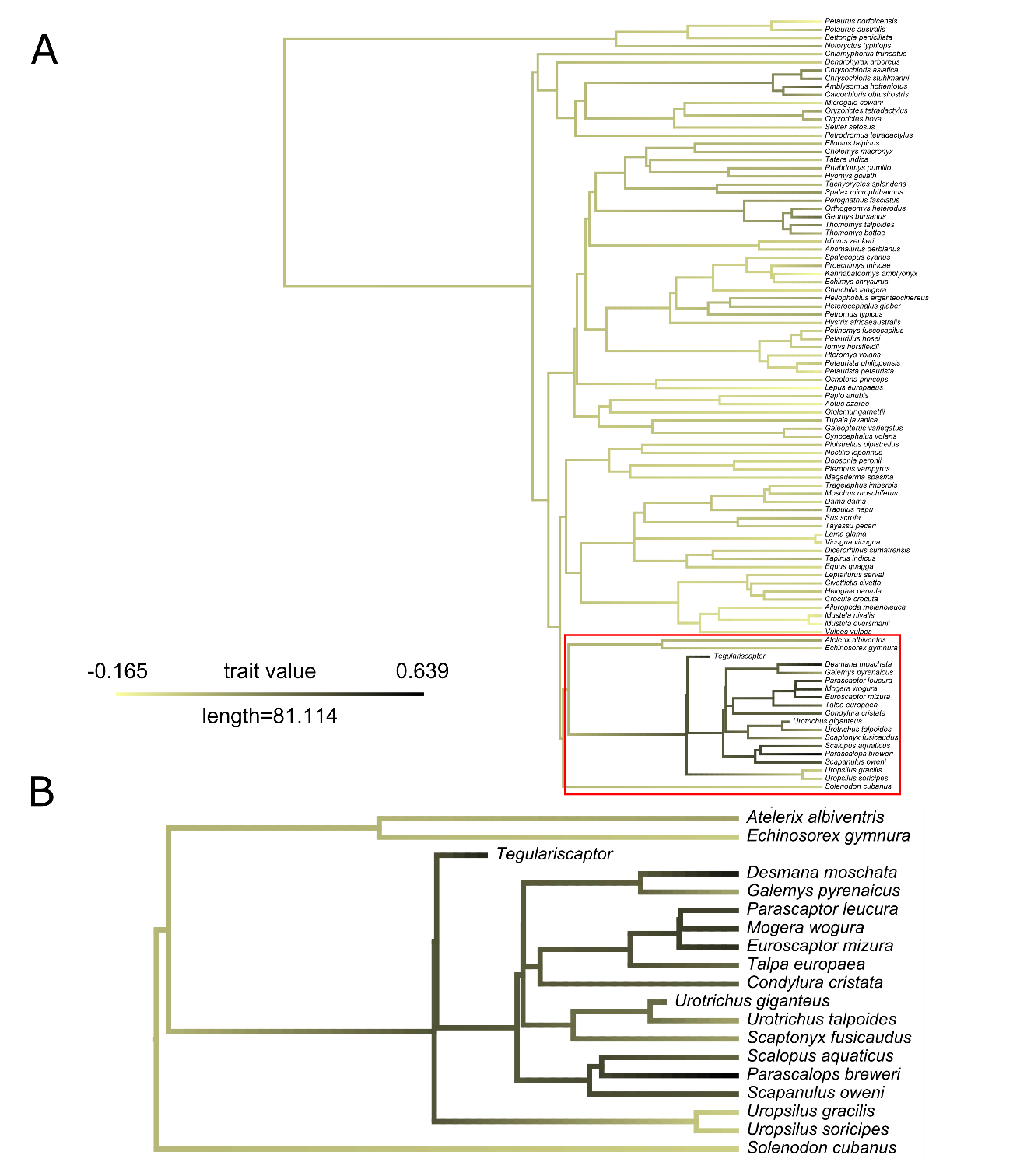
**

Fig. S2.

Phylogenetic mapping of the humeral head bone fraction (BV/TV). Given its correlation with body size, the ‘size-corrected’ residuals (BV/TV ~ body mass) were plotted. *(A)* Whole tree of sampled species. *(B)* Focus on the family Talpidae. The reconstruction was obtained with a Brownian motion maximum likelihood algorithm (package phytools, function *contmap*; Revell, 2012). Here the Tree 1A is used (see Methods, main text). Note that even though a reversion to lower values is reconstructed for *Uropsilus*, it is more parsimonious to acknowledge one single acquisition of an increased BV/TV at the base of non-uropsiline talpids.


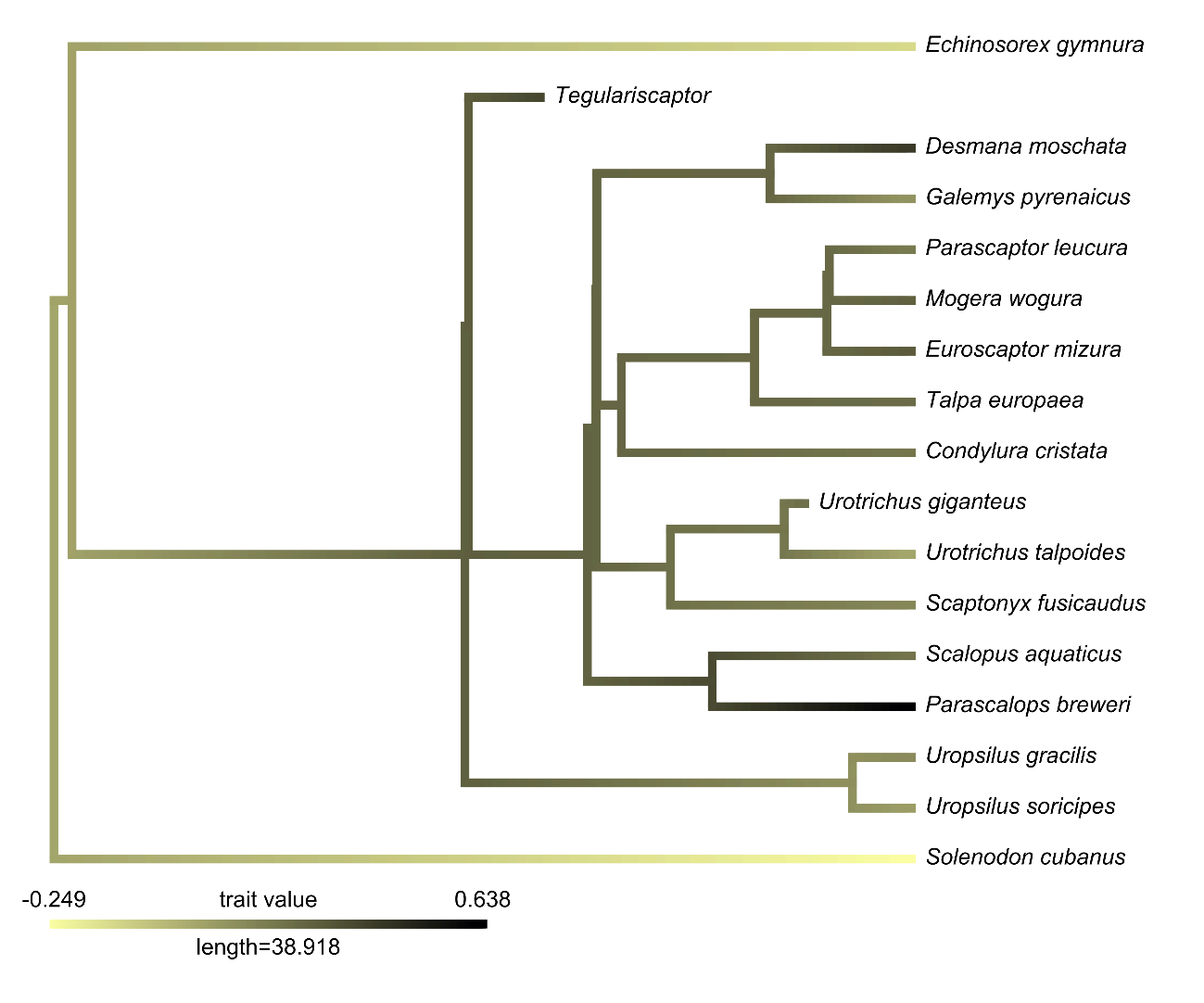


Fig. S3.

Phylogenetic mapping of the humeral capitulum bone fraction (BV/TV). Given its correlation with body size, the ‘size-corrected’ residuals (BV/TV ~ body mass) were plotted. The reconstruction was obtained with a Brownian motion maximum likelihood algorithm (package phytools, function *contmap*; Revell, 2012). Here the Tree 1A is used (see Methods, main text). Note that even though a reversion to lower values is reconstructed for *Uropsilus*, it is more parsimonious to acknowledge one single acquisition of an increased BV/TV at the base of non-uropsiline talpids.

**
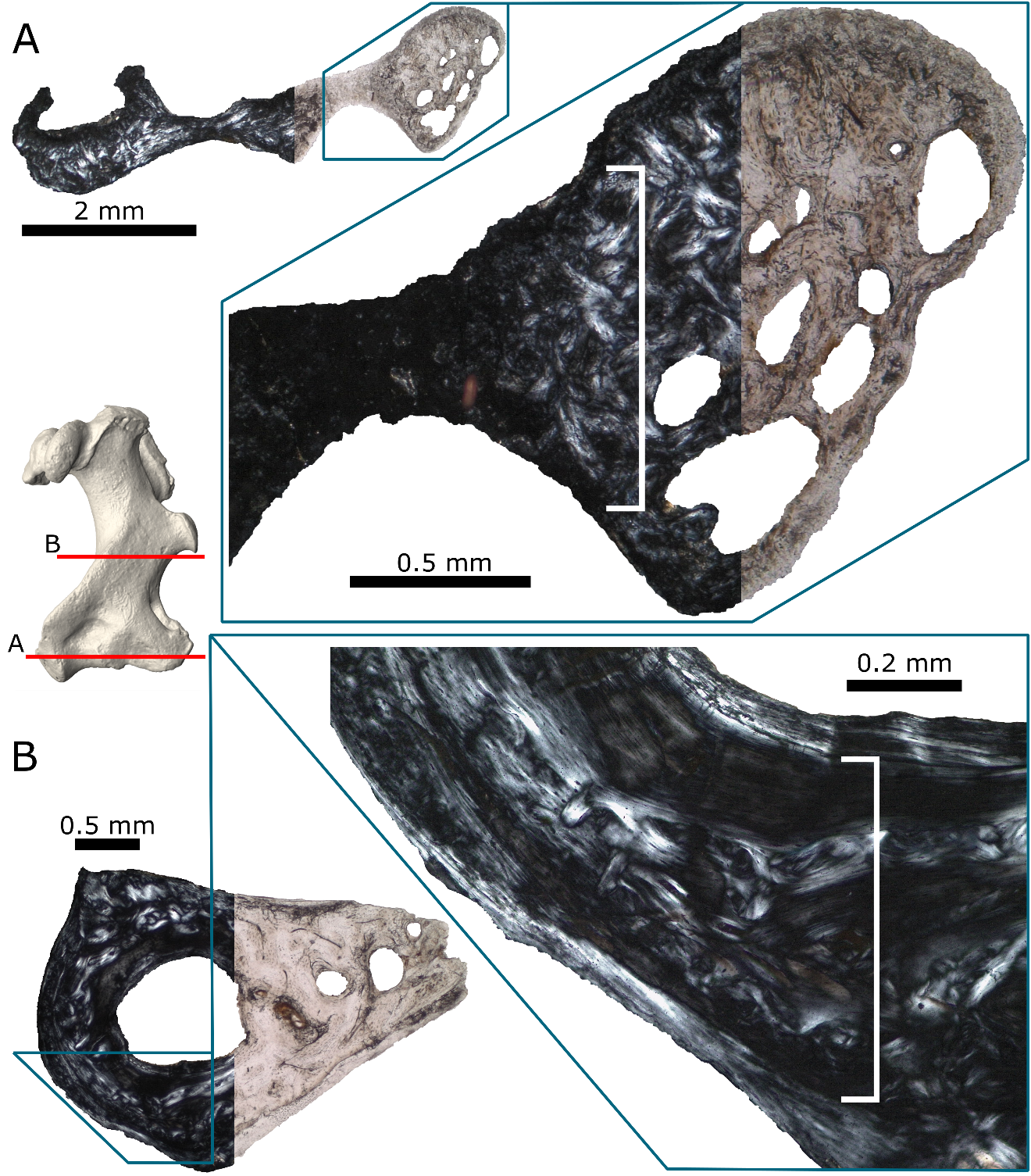
**

Fig. S4.

Talpid (true moles) bone histology. All thin-sections are displayed under normal transmitted light (right) and cross-polarized light (left). The sections are made *(A)* at the level of the distal epiphysis and *(B)* close to mid-diaphysis. White brackets indicate the extent of coarse cancellous bone (CCCB). The three-dimensional rendering with approximate position of the thin-sections is not to scale. Specimen: *Tegulariscaptor minor*, SMNS-P- 47918 (Staatliches Museum für Naturkunde Stuttgart)


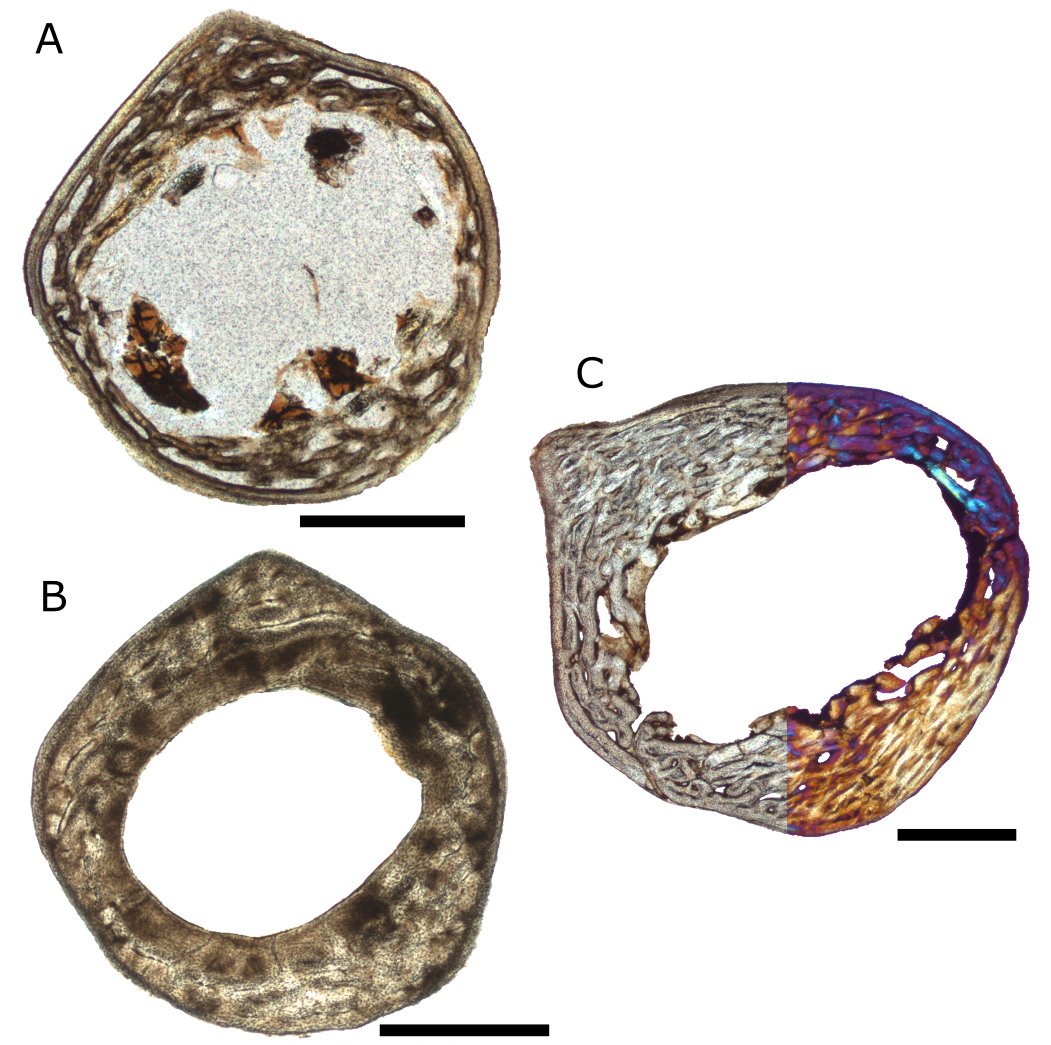


Fig. S5.

Extant talpine humeral bone histology at mid-diaphysis. *Condylura cristata* cross-sections under normal transmitted light of *(A)* a subadult specimen (PIMUZ A/V 6310; Palaeontological Institute and Museum, Univ. of Zurich) and *(B)* an adult specimen (PIMUZ A/V 6317.). *(C)* Adult *Talpa europaea* (NMB C.1454 (Naturhistorisches Museum Basel) thin-section observed under normal transmitted light (left) and cross-polarized light with Lambda compensator (right). Note the loose trabeculae in the subdadult specimen, and the compacted coarse cancellous bone found in at a more mature stage. Scale bars = 1 mm.


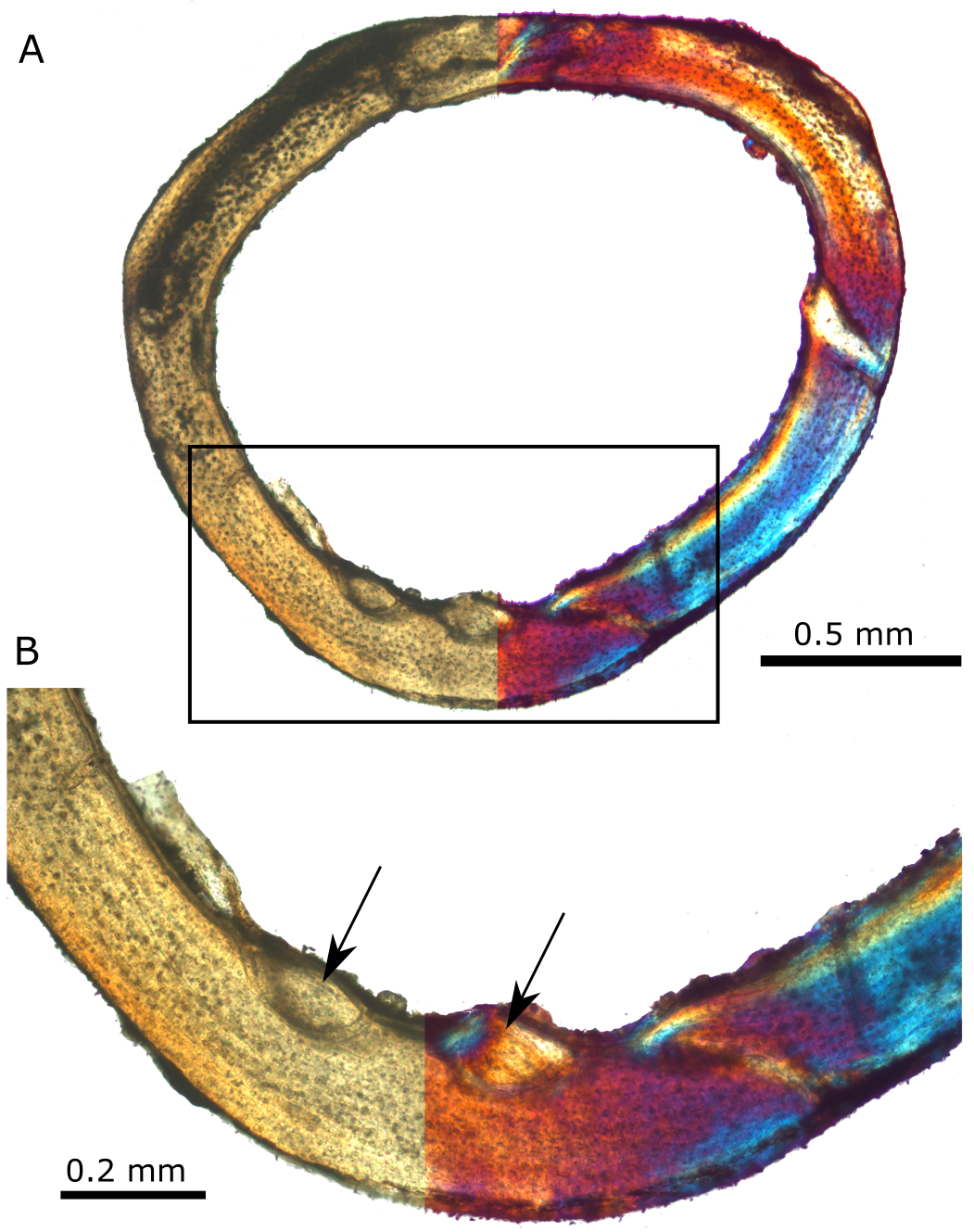


Fig. S6.

Bone histology of the talpid femur at mid-diaphysis. *(A)* Whole thin-section; *(B)* Detail of previous showing small areas potentially corresponding to compacted coarse cancellous bone (arrows). Thin-sections are displayed under normal transmitted light (left) and cross-polarized light with Lambda compensator (right). Specimen *Talpa europaea*, PIMUZ A/V 6310 (Palaeontological Institute and Museum, Univ. of Zurich).

**
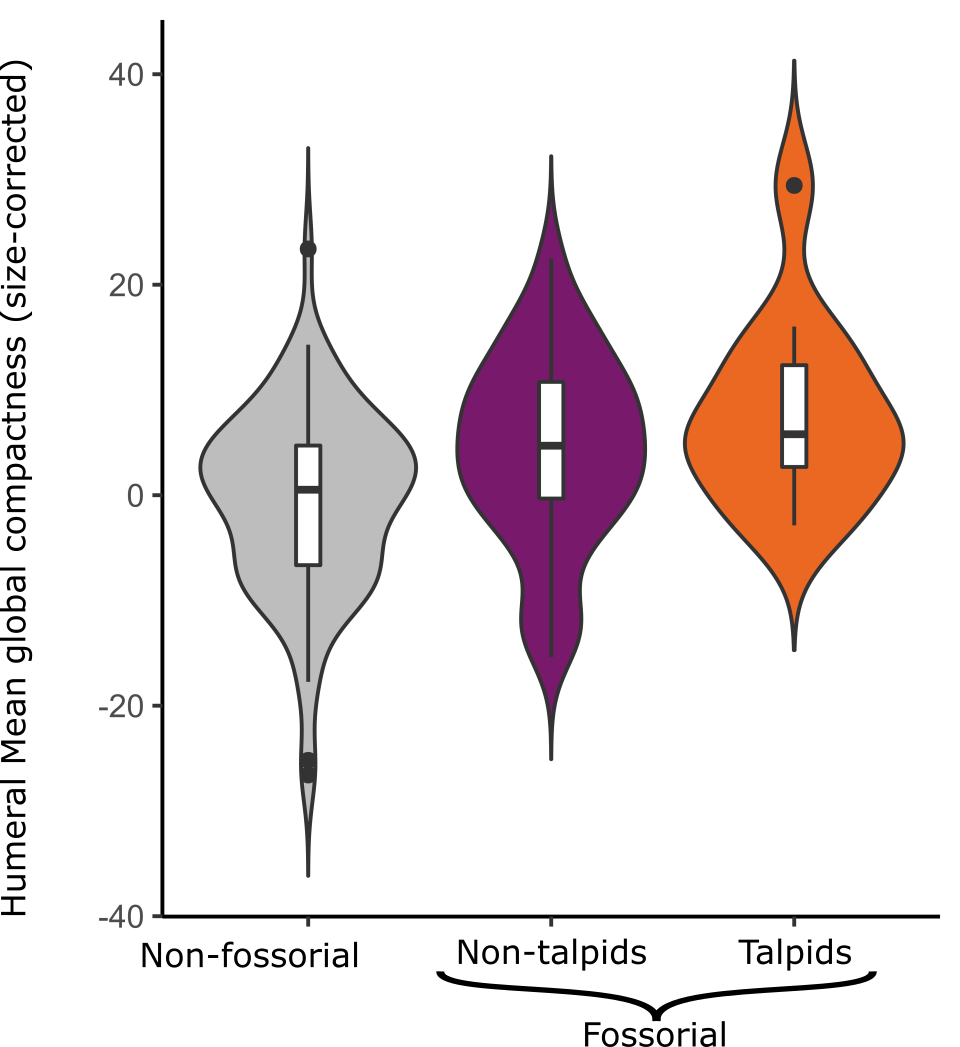
**

Fig. S7.

Mean global compactness of the humeral diaphysis among fossorial talpids (true moles), other subterranean species, and non-subterranean mammals. Given its correlation with body size, the ‘size-corrected’ residuals (mean global compactness~ body mass) were plotted.


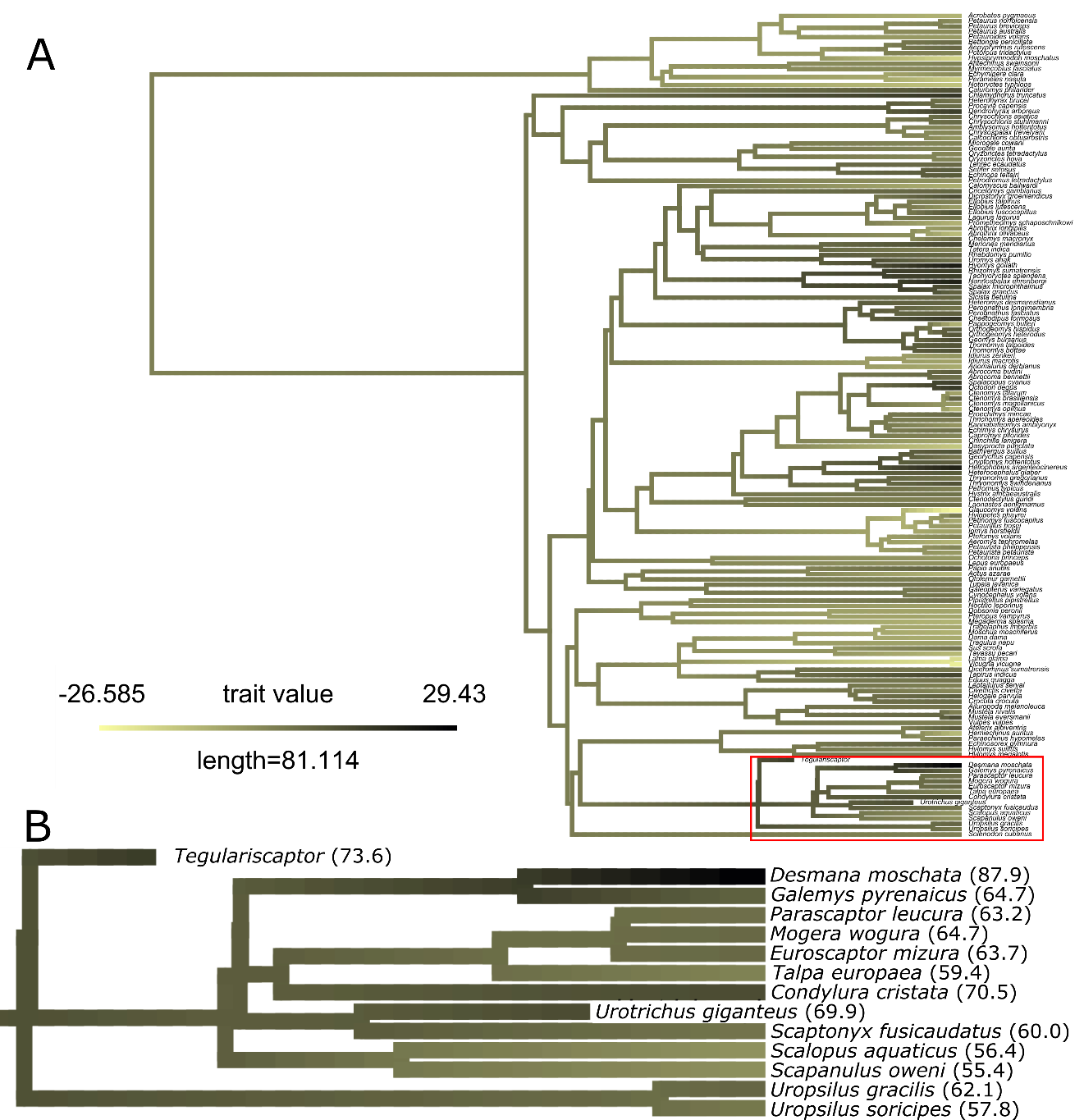


Fig. S8.

Phylogenetic mapping of the mean global compactness of the humeral diaphysis. Given its correlation with body size, the ‘size-corrected’ residuals (mean global compactness~ body mass) were plotted. (A) Whole tree of sampled species. (B) Focus on the family Talpidae. The reconstruction was obtained with a Brownian motion maximum likelihood algorithm (package phytools, function contmap (1)). Approximate raw compactness values (%) are between brackets. Here the Tree 1A is used (see Methods, main text).


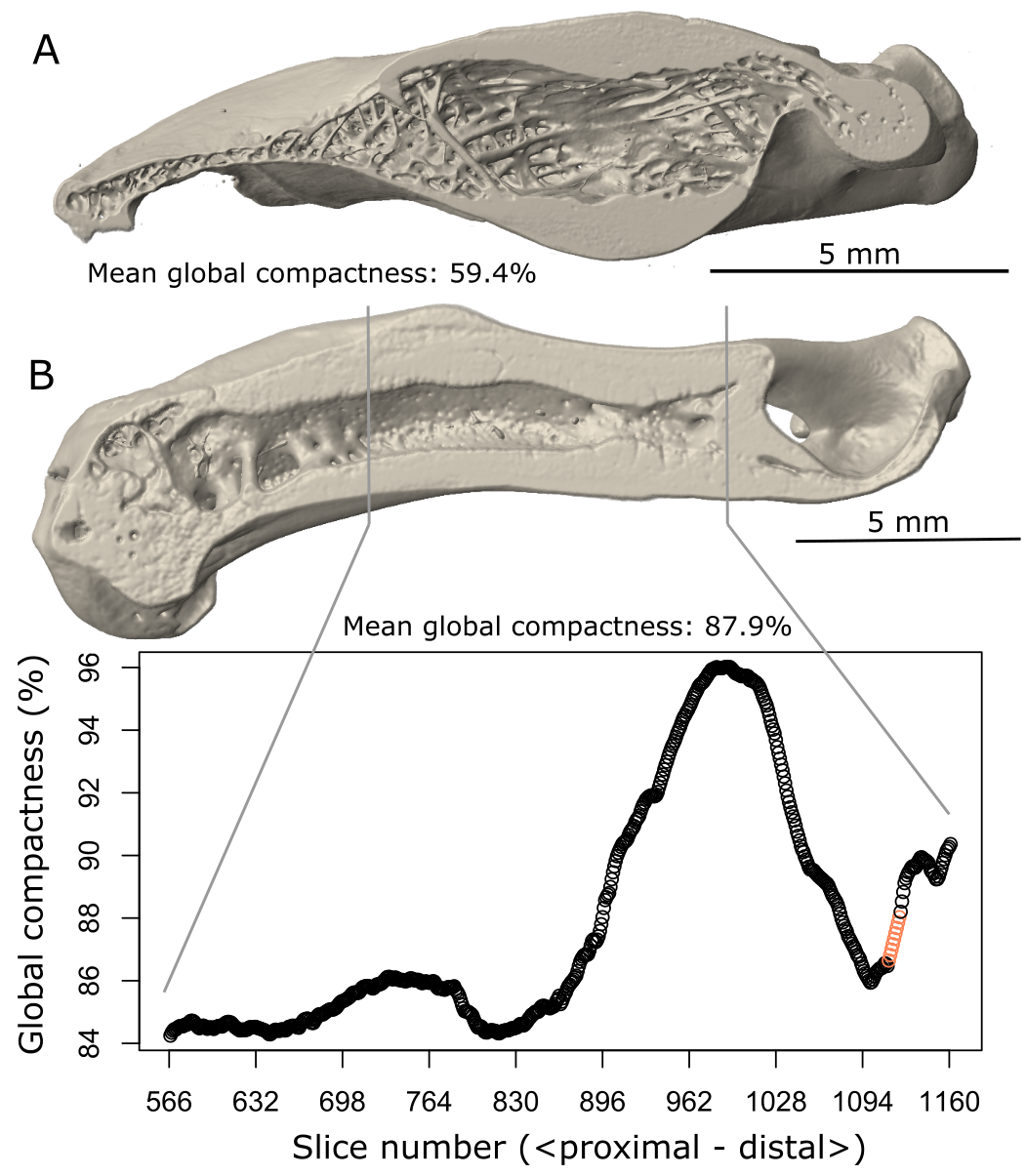


**Fig. S9.**

Humerus diaphysis structure among talpids. *(A)* 3D rendering of European mole (*Talpa europaea*, ZMB_Mam_60682). *(B)* 3D rendering and proximodistal profile of the mean global compactness of Russian desman (*Desmana moschata*, SMNS-Z-MAM-160). In both cases the 3D rendering is a medial view of the lateral half of the humerus. Orange circles in the profile denote a region for which the parameter was estimated based on neighboring values (see *(47)*).


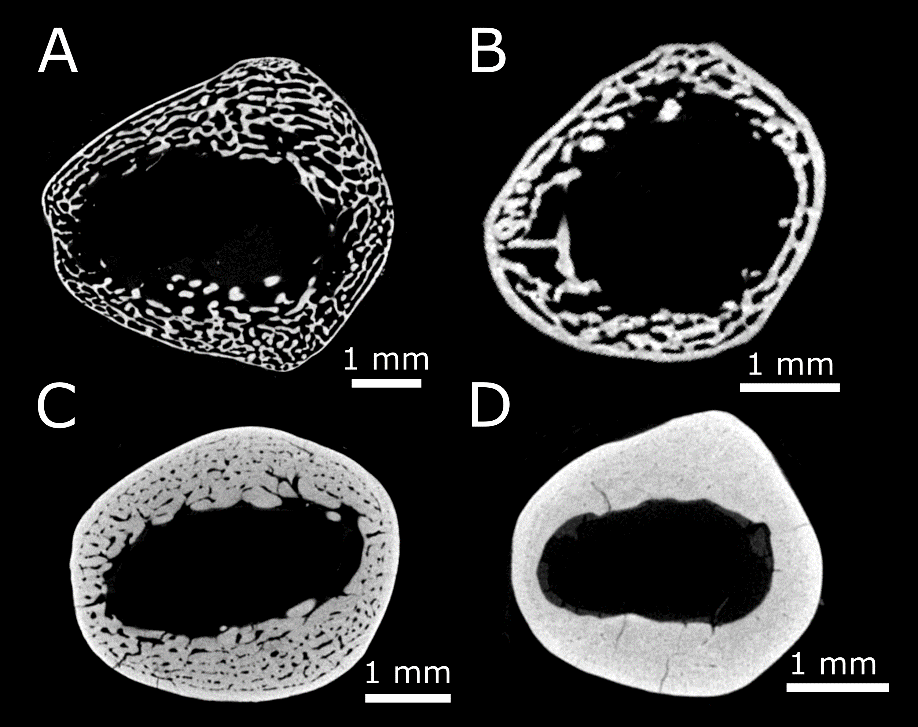


Fig. S10.

Compacted coarse cancellous bone (CCCB) at several stages of osteogenesis in the humeral mid-diaphysis of talpids. *(A) Scalopus aquaticus* (NMW21387), *(B)* *Condylura cristata* (SMNS-Z-MAM-32943), *(C)* *Parascalops breweri* (NMW62569). *(D)* *Euroscaptor mizura* ZMB_Mam_103981). Note the progressive infilling of trabecular spaces from subadult to adult stages. All cross-sections are virtual (obtained through computed tomography) and made at humeral mid-diaphysis.

Table S1.

Correlation between humeral head bone fraction / trabecular thickness and subterranean lifestyle. Phylogenetically informed ANCOVA output, where body mass is used as a covariate, using a model adjusted with the optimized Pagel’s lambda (see Material and Methods), and accounting for species variance heterogeneity (non-ultrametric timetree). Multiple phylogenetic trees were used to account for topology uncertainty (see Methods, main text).

| **Humeral head bone fraction** | | | | |
| --- | --- | --- | --- | --- |
|  | Value | Std.Error | t-value | p-value |
| Tree 1A |  |  |  |  |
| (Intercept) | 0.22275931 | 0.0662539 | 3.362207 | 1.13E-03 |
| Lifestyle | 0.18134334 | 0.02960053 | 6.126355 | 2.20E-08 |
| Log Body Mass | 0.05432591 | 0.01370411 | 3.964206 | 1.45E-04 |
| Tree 1B |  |  |  |  |
| (Intercept) | 0.22335934 | 0.06669113 | 3.349161 | 1.18E-03 |
| Lifestyle | 0.17782865 | 0.02966576 | 5.994407 | 3.95E-08 |
| Log Body Mass | 0.05422983 | 0.01368576 | 3.962501 | 1.46E-04 |
| Tree 2 |  |  |  |  |
| (Intercept) | 0.22312433 | 0.06735034 | 3.312891 | 1.32E-03 |
| Lifestyle | 0.17876031 | 0.02969183 | 6.020522 | 3.52E-08 |
| Log Body Mass | 0.05438426 | 0.01372935 | 3.961168 | 1.47E-04 |
|  |  |  |  |  |
| **Trabecular thickness** | | | | |
| Tree 1A |  |  |  |  |
| (Intercept) | -1.4907737 | 0.06045188 | -24.660501 | 2.54E-42 |
| Lifestyle | 0.2133527 | 0.03827409 | 5.574337 | 2.46E-07 |
| Log Body Mass | 0.2166043 | 0.01813065 | 11.946859 | 2.05E-20 |
| Tree 1B |  |  |  |  |
| (Intercept) | -1.4908126 | 0.0610332 | -24.42626 | 5.44E-42 |
| Lifestyle | 0.2124808 | 0.03839428 | 5.53418 | 2.93E-07 |
| Log Body Mass | 0.2166318 | 0.0181726 | 11.92079 | 2.32E-20 |
| Tree 2 |  |  |  |  |
| (Intercept) | -1.4907265 | 0.06027259 | -24.733075 | 2.01E-42 |
| Lifestyle | 0.2137187 | 0.03823224 | 5.590012 | 2.30E-07 |
| Log Body Mass | 0.2165952 | 0.01811824 | 11.954538 | 1.98E-20 |

Table S2.

Correlation between mid-lumbar vertebra centrum bone fraction and subterranean lifestyle. Phylogenetically informed ANCOVA output, where body mass is used as a covariate, using a model adjusted with the optimized Pagel’s lambda (see Material and Methods) and accounting for species variance heterogeneity (non-ultrametric timetree).

| **Mid-lumbar centrum bone fraction** | | | | |
| --- | --- | --- | --- | --- |
|  | Value | Std.Error | t-value | p-value |
| Tree 1A |  |  |  |  |
| (Intercept) | -1.13883347 | 0.11589459 | -9.826459 | 3.63E-18 |
| Lifestyle | 0.01959634 | 0.05862611 | 0.3342596 | 7.39E-01 |
| Log Body Mass | 0.1412363 | 0.02603728 | 5.4243883 | 2.08E-07 |
| Tree 1B |  |  |  |  |
| (Intercept) | -1.13883347 | 0.11589459 | -9.826459 | 3.63E-18 |
| Lifestyle | 0.01959634 | 0.05862611 | 0.3342596 | 7.39E-01 |
| Log Body Mass | 0.1412363 | 0.02603728 | 5.4243883 | 2.08E-07 |
| Tree 2 |  |  |  |  |
| (Intercept) | -1.1382586 | 0.12085775 | -9.4181679 | 4.53E-17 |
| Lifestyle | 0.02200792 | 0.0590971 | 0.3724028 | 7.10E-01 |
| Log Body Mass | 0.14121592 | 0.02630239 | 5.3689383 | 2.71E-07 |

Table S3.

Correlation between humeral diaphysis mean global compactness and subterranean lifestyle. Phylogenetically informed ANCOVA output, where body mass is used as a covariate, using a model adjusted with the optimized Pagel’s lambda (see Material and Methods), and accounting for species variance heterogeneity (non-ultrametric timetree).

| **Humeral diaphysis mean humeral compactness** | | | | |
| --- | --- | --- | --- | --- |
|  | Value | Std.Error | t-value | p-value |
| Tree 1A |  |  |  |  |
| (Intercept) | 52.394915 | 5.2291072 | 10.019859 | 1.85E-18 |
| Lifestyle | 2.602484 | 1.912816 | 1.360551 | 1.76E-01 |
| Log Body Mass | 2.087752 | 0.9578568 | 2.179608 | 3.08E-02 |
| Tree 1B |  |  |  |  |
| (Intercept) | 52.389564 | 5.232021 | 10.013256 | 1.93E-18 |
| Lifestyle | 2.542601 | 1.916809 | 1.326476 | 1.87E-01 |
| Log Body Mass | 2.083853 | 0.957923 | 2.175387 | 3.11E-02 |
| Tree 2 |  |  |  |  |
| (Intercept) | 52.373772 | 5.2720829 | 9.934171 | 3.12E-18 |
| Lifestyle | 2.468946 | 1.9164051 | 1.288322 | 2.00E-01 |
| Log Body Mass | 2.094757 | 0.9587111 | 2.184972 | 3.04E-02 |

Table S4.

Lifestyle classification for the sampled species.

| Order | Family | Lifestyle |  | Lineage |  | Taxon |
| --- | --- | --- | --- | --- | --- | --- |
| Afrosoricida | Chrysochloridae | Su |  | Su_Chry |  | *Chrysospalax trevelyani* |
| Afrosoricida | Chrysochloridae | Su |  | Su_Chry |  | *Chrysochloris stuhlmanni* |
| Afrosoricida | Chrysochloridae | Su |  | Su_Chry |  | *Amblysomus hottentotus* |
| Afrosoricida | Chrysochloridae | Su |  | Su_Chry |  | *Chrysochloris asiatica* |
| Afrosoricida | Chrysochloridae | Su |  | Su_Chry |  | *Calcochloris obtusirostris* |
| Afrosoricida | Tenrecidae | Su |  | Su_Ory |  | *Oryzorictes hova* |
| Afrosoricida | Tenrecidae | Su |  | Su_Ory |  | *Oryzorictes tetradactylus* |
| Afrosoricida | Tenrecidae | Te |  | Out_Su_Chry_Ory |  | *Geogale aurita* |
| Afrosoricida | Tenrecidae | Te |  | Out_Su_Chry_Ory |  | *Tenrec ecaudatus* |
| Afrosoricida | Tenrecidae | Te |  | Out_Su_Chry_Ory |  | *Setifer setosus* |
| Afrosoricida | Tenrecidae | Te |  | Out_Su_Chry_Ory |  | *Echinops telfairi* |
| Afrosoricida | Tenrecidae | Te |  | Out_Su_Chry_Ory |  | *Microgale cowani* |
| Artiodactyla | Bovidae | Te |  | Else |  | *Tragelaphus imberbis* |
| Artiodactyla | Bovidae | Te |  | Else |  | *Kobus ellipsiprymnus* |
| Artiodactyla | Bovidae | Te |  | Else |  | *Cephalophus silvicultor* |
| Artiodactyla | Camelidae | Te |  | Else |  | *Lama glama* |
| Artiodactyla | Camelidae | Te |  | Else |  | *Vicugna vicugna* |
| Artiodactyla | Cervidae | Te |  | Else |  | *Dama dama* |
| Artiodactyla | Giraffidae | Te |  | Else |  | *Okapia johnstoni* |
| Artiodactyla | Moschidae | Te |  | Else |  | *Moschus moschiferus* |
| Artiodactyla | Suidae | Te |  | Else |  | *Sus scrofa* |
| Artiodactyla | Tayassuidae | Te |  | Else |  | *Tayassu pecari* |
| Artiodactyla | Tragulidae | Te |  | Else |  | *Tragulus napu* |
| Carnivora | Canidae | Te |  | Out_Ae_Chi |  | *Vulpes vulpes* |
| Carnivora | Canidae | Te |  | Out_Ae_Chi |  | *Canis lupus* |
| Carnivora | Felidae | Te |  | Out_Ae_Chi |  | *Leptailurus serval* |
| Carnivora | Felidae | Te |  | Out_Ae_Chi |  | *Acinonyx jubatus* |
| Carnivora | Herpestidae | Te |  | Out_Ae_Chi |  | *Helogale parvula* |
| Carnivora | Hyaenidae | Te |  | Out_Ae_Chi |  | *Crocuta crocuta* |
| Carnivora | Mustelidae | Te |  | Out_Ae_Chi |  | *Mustela eversmanii* |
| Carnivora | Mustelidae | Te |  | Out_Ae_Chi |  | *Mustela nivalis* |
| Carnivora | Ursidae | Te |  | Out_Ae_Chi |  | *Ailuropoda melanoleuca* |
| Carnivora | Viverridae | Te |  | Out_Ae_Chi |  | *Civettictis civetta* |
| Chiroptera | Megadermatidae | Ae |  | Ae_Chi |  | *Megaderma spasma* |
| Chiroptera | Noctilionidae | Ae |  | Ae_Chi |  | *Noctilio leporinus* |
| Chiroptera | Pteropodidae | Ae |  | Ae_Chi |  | *Dobsonia peronii* |
| Chiroptera | Pteropodidae | Ae |  | Ae_Chi |  | *Pteropus vampyrus* |
| Chiroptera | Pteropodidae | Ae |  | Ae_Chi |  | *Pteropus giganteus* |
| Chiroptera | Vespertilionidae | Ae |  | Ae_Chi |  | *Pipistrellus pipistrellus* |
| Cingulata | Dasypodidae | Su |  | Su_Xen |  | *Chlamyphorus truncatus* |
| Dasyuromorphia | Dasyuridae | Te |  | Else |  | *Antechinus swainsonii* |
| Dasyuromorphia | Myrmecobiidae | Te |  | Else |  | *Myrmecobius fasciatus* |
| Dermoptera | Cynocephalidae | Ae |  | Ae_Der |  | *Galeopterus variegatus* |
| Dermoptera | Cynocephalidae | Ae |  | Ae_Der |  | *Cynocephalus volans* |
| Didelphimorphia | Didelphidae | Te |  | Else |  | *Caluromys philander* |
| Diprotodontia | Acrobatidae | Ae |  | Ae_Mars |  | *Acrobates pygmaeus* |
| Diprotodontia | Petauridae | Ae |  | Ae_Mars |  | *Petaurus australis* |
| Diprotodontia | Petauridae | Ae |  | Ae_Mars |  | *Petaurus norfolcensis* |
| Diprotodontia | Petauridae | Ae |  | Ae_Mars |  | *Petaurus breviceps* |
| Diprotodontia | Pseudocheiridae | Ae |  | Ae_Mars |  | *Petauroides volans* |
| Diprotodontia | Hypsiprymnodontidae | Te |  | Out_Ae_Mars |  | *Hypsiprymnodon moschatus* |
| Diprotodontia | Potoroidae | Te |  | Out_Ae_Mars |  | *Potorous tridactylus* |
| Diprotodontia | Potoroidae | Te |  | Out_Ae_Mars |  | *Bettongia penicillata* |
| Diprotodontia | Potoroidae | Te |  | Out_Ae_Mars |  | *Aepyprymnus rufescens* |
| Diprotodontia | Potoroidae | Te |  | Out_Ae_Mars |  | *Bettongia lesueur* |
| Diprotodontia | Potoroidae | Te |  | Out_Ae_Mars |  | *Bettongia gaimardi* |
| Eulipotyphla | Talpidae | Su |  | Su_Tal |  | *Scaptonyx fusicaudus* |
| Eulipotyphla | Talpidae | Su |  | Su_Tal |  | *Scapanulus oweni* |
| Eulipotyphla | Talpidae | Su |  | Su_Tal |  | *Parascaptor leucura* |
| Eulipotyphla | Talpidae | Su |  | Su_Tal |  | *Scalopus aquaticus* |
| Eulipotyphla | Talpidae | Su |  | Su_Tal |  | *Urotrichus giganteus* |
| Eulipotyphla | Talpidae | Su |  | Su_Tal |  | *Tegulariscaptor* |
| Eulipotyphla | Talpidae | Su |  | Su_Tal |  | *Euroscaptor mizura* |
| Eulipotyphla | Talpidae | Su |  | Su_Tal |  | *Talpa europaea* |
| Eulipotyphla | Talpidae | Su |  | Su_Tal |  | *Mogera wogura* |
| Eulipotyphla | Talpidae | Su |  | Su_Tal |  | *Parascalops breweri* |
| Eulipotyphla | Talpidae | Su* |  | Su_Tal |  | *Galemys pyrenaicus* |
| Eulipotyphla | Talpidae | Su* |  | Su_Tal |  | *Condylura cristata* |
| Eulipotyphla | Talpidae | Su* |  | Su_Tal |  | *Desmana moschata* |
| Eulipotyphla | Erinaceidae | Te |  | Out_Su_Tal |  | *Hylomys suillus* |
| Eulipotyphla | Erinaceidae | Te |  | Out_Su_Tal |  | *Hylomys megalotis* |
| Eulipotyphla | Erinaceidae | Te |  | Out_Su_Tal |  | *Paraechinus hypomelas* |
| Eulipotyphla | Erinaceidae | Te |  | Out_Su_Tal |  | *Hemiechinus auritus* |
| Eulipotyphla | Erinaceidae | Te |  | Out_Su_Tal |  | *Atelerix albiventris* |
| Eulipotyphla | Erinaceidae | Te |  | Out_Su_Tal |  | *Echinosorex gymnura* |
| Eulipotyphla | Solenodontidae | Te |  | Else |  | *Solenodon cubanus* |
| Eulipotyphla | Talpidae | Te |  | Out_Su_Tal |  | *Uropsilus gracilis* |
| Eulipotyphla | Talpidae | Te |  | Out_Su_Tal |  | *Uropsilus soricipes* |
| Hyracoidea | Procaviidae | Te |  | Out_Su_Xen |  | *Dendrohyrax arboreus* |
| Hyracoidea | Procaviidae | Te |  | Out_Su_Xen |  | *Heterohyrax brucei* |
| Hyracoidea | Procaviidae | Te |  | Out_Su_Xen |  | *Procavia capensis* |
| Lagomorpha | Leporidae | Te |  | Else |  | *Lepus europaeus* |
| Lagomorpha | Leporidae | Te |  | Else |  | *Ochotona princeps* |
| Lagomorpha | Leporidae | Te |  | Else |  | *Ochotona rufescens* |
| Macroscelidea | Macroscelididae | Te |  | Else |  | *Petrodromus tetradactylus* |
| Notoryctemorphia | Notoryctidae | Su |  | Su_Noto |  | *Notoryctes typhlops* |
| Peramelemorphia | Peramelidae | Te |  | Out_Su_Noto |  | *Perameles nasuta* |
| Peramelemorphia | Peramelidae | Te |  | Out_Su_Noto |  | *Echymipera clara* |
| Perissodactyla | Equidae | Te |  | Out_Ae_Chi |  | *Equus quagga* |
| Perissodactyla | Rhinocerotidae | Te |  | Out_Ae_Chi |  | *Dicerorhinus sumatrensis* |
| Perissodactyla | Rhinocerotidae | Te |  | Out_Ae_Chi |  | *Diceros bicornis* |
| Perissodactyla | Tapiridae | Te |  | Out_Ae_Chi |  | *Tapirus indicus* |
| Primates | Aotidae | Te |  | Out_Ae_Der |  | *Aotus azarae* |
| Primates | Cercopithecidae | Te |  | Out_Ae_Der |  | *Papio anubis* |
| Primates | Galagidae | Te |  | Out_Ae_Der |  | *Otolemur garnettii* |
| Primates | Hominidae | Te |  | Out_Ae_Der |  | *Pan troglodytes* |
| Proboscidea | Elephantidae | Te |  | Else |  | *Elephas maximus* |
| Rodentia | Anomaluridae | Ae |  | Ae_Ano |  | *Idiurus zenkeri* |
| Rodentia | Anomaluridae | Ae |  | Ae_Ano |  | *Idiurus macrotis* |
| Rodentia | Anomaluridae | Ae |  | Ae_Ano |  | *Anomalurus derbianus* |
| Rodentia | Anomaluridae | Ae |  | Ae_Ano |  | *Anomalurus beecrofti* |
| Rodentia | Sciuridae | Ae |  | Ae_Sciu |  | *Glaucomys volans* |
| Rodentia | Sciuridae | Ae |  | Ae_Sciu |  | *Petaurillus hosei* |
| Rodentia | Sciuridae | Ae |  | Ae_Sciu |  | *Iomys horsfieldii* |
| Rodentia | Sciuridae | Ae |  | Ae_Sciu |  | *Aeromys tephromelas* |
| Rodentia | Sciuridae | Ae |  | Ae_Sciu |  | *Petinomys fuscocapilus* |
| Rodentia | Sciuridae | Ae |  | Ae_Sciu |  | *Hylopetes phayrei* |
| Rodentia | Sciuridae | Ae |  | Ae_Sciu |  | *Petaurista petaurista* |
| Rodentia | Sciuridae | Ae |  | Ae_Sciu |  | *Pteromys volans* |
| Rodentia | Sciuridae | Ae |  | Ae_Sciu |  | *Petaurista philippensis* |
| Rodentia | Sciuridae | Ae |  | Ae_Sciu |  | *Eoglaucomys fimbriatus* |
| Rodentia | Bathyergidae | Su |  | Su_Hete |  | *Georychus capensis* |
| Rodentia | Bathyergidae | Su |  | Su_Hete |  | *Geomys bursarius* |
| Rodentia | Bathyergidae | Su |  | Su_Hete |  | *Cryptomys hottentotus* |
| Rodentia | Bathyergidae | Su |  | Su_Hete |  | *Heliophobius argenteocinereus* |
| Rodentia | Bathyergidae | Su |  | Su_Hete |  | *Bathyergus suillus* |
| Rodentia | Bathyergidae | Su |  | Su_Hete |  | *Heterocephalus glaber* |
| Rodentia | Cricetidae | Su |  | Su_Prom |  | *Prometheomys schaposchnikowi* |
| Rodentia | Cricetidae | Su |  | Su_Ello |  | *Ellobius fuscocapillus* |
| Rodentia | Cricetidae | Su |  | Su_Ello |  | *Ellobius lutescens* |
| Rodentia | Cricetidae | Su |  | Su_Noti |  | *Chelemys macronyx* |
| Rodentia | Cricetidae | Su |  | Su_Ello |  | *Ellobius talpinus* |
| Rodentia | Ctenomyidae | Su |  | Su_Cten |  | *Ctenomys brasiliensis* |
| Rodentia | Ctenomyidae | Su |  | Su_Cten |  | *Ctenomys opimus* |
| Rodentia | Ctenomyidae | Su |  | Su_Cten |  | *Ctenomys talarum* |
| Rodentia | Ctenomyidae | Su |  | Su_Cten |  | *Ctenomys magellanicus* |
| Rodentia | Ctenomyidae | Su |  | Su_Cten |  | *Ctenomys tucumanus* |
| Rodentia | Geomyidae | Su |  | Su_Geo |  | *Pappogeomys bulleri* |
| Rodentia | Geomyidae | Su |  | Su_Geo |  | *Orthogeomys hispidus* |
| Rodentia | Geomyidae | Su |  | Su_Geo |  | *Thomomys talpoides* |
| Rodentia | Geomyidae | Su |  | Su_Geo |  | *Thomomys bottae* |
| Rodentia | Geomyidae | Su |  | Su_Geo |  | *Orthogeomys heterodus* |
| Rodentia | Octodontidae | Su |  | Su_Octo |  | *Spalacopus cyanus* |
| Rodentia | Spalacidae | Su |  | Su_Spal |  | *Spalax graecus* |
| Rodentia | Spalacidae | Su |  | Su_Spal |  | *Nannospalax ehrenbergi* |
| Rodentia | Spalacidae | Su |  | Su_Spal |  | *Rhizomys sumatrensis* |
| Rodentia | Spalacidae | Su |  | Su_Spal |  | *Tachyoryctes splendens* |
| Rodentia | Spalacidae | Su |  | Su_Spal |  | *Spalax microphthalmus* |
| Rodentia | Abrocomidae | Te |  | Else |  | *Abrocoma budini* |
| Rodentia | Abrocomidae | Te |  | Else |  | *Abrocoma bennettii* |
| Rodentia | Calomyscidae | Te |  | Out_SciuAnoSpalProm |  | *Calomyscus bailwardi* |
| Rodentia | Capromyidae | Te |  | Out_Su_Cten |  | *Capromys pilorides* |
| Rodentia | Chinchillidae | Te |  | Else |  | *Chinchilla lanigera* |
| Rodentia | Cricetidae | Te |  | Out_Su_Noti |  | *Abrothrix olivaceus* |
| Rodentia | Cricetidae | Te |  | Out_Su_Ello |  | *Lagurus lagurus* |
| Rodentia | Cricetidae | Te |  | Out_Su_Noti |  | *Abrothrix longipilis* |
| Rodentia | Cricetidae | Te |  | Out_SciuAnoSpalProm |  | *Dicrostonyx groenlandicus* |
| Rodentia | Ctenodactylidae | Te |  | Else |  | *Ctenodactylus gundi* |
| Rodentia | Dasyproctidae | Te |  | Else |  | *Dasyprocta punctata* |
| Rodentia | Diatomyidae | Te |  | Else |  | *Laonastes aenigmamus* |
| Rodentia | Diatomyidae | Te |  | Else |  | *Dolichotis patagonum* |
| Rodentia | Dipodidae | Te |  | Else |  | *Sicista betulina* |
| Rodentia | Echimyidae | Te |  | Out_Su_Cten |  | *Proechimys mincae* |
| Rodentia | Echimyidae | Te |  | Out_Su_Cten |  | *Kannabateomys amblyonyx* |
| Rodentia | Echimyidae | Te |  | Out_Su_Cten |  | *Thrichomys apereoides* |
| Rodentia | Echimyidae | Te |  | Out_Su_Cten |  | *Echimys chrysurus* |
| Rodentia | Heteromyidae | Te |  | Out_Su_Geo |  | *Chaetodipus formosus* |
| Rodentia | Heteromyidae | Te |  | Out_Su_Geo |  | *Perognathus longimembris* |
| Rodentia | Heteromyidae | Te |  | Out_Su_Geo |  | *Heteromys desmarestianus* |
| Rodentia | Heteromyidae | Te |  | Out_Su_Geo |  | *Perognathus fasciatus* |
| Rodentia | Hystricidae | Te |  | Else |  | *Hystrix africaeaustralis* |
| Rodentia | Muridae | Te |  | Out_SciuAnoSpalProm |  | *Hyomys goliath* |
| Rodentia | Muridae | Te |  | Out_SciuAnoSpalProm |  | *Uromys anak* |
| Rodentia | Muridae | Te |  | Out_SciuAnoSpalProm |  | *Meriones meridianus* |
| Rodentia | Muridae | Te |  | Out_SciuAnoSpalProm |  | *Tatera indica* |
| Rodentia | Muridae | Te |  | Out_SciuAnoSpalProm |  | *Rhabdomys pumilio* |
| Rodentia | Nesomyidae | Te |  | Out_SciuAnoSpalProm |  | *Cricetomys gambianus* |
| Rodentia | Octodontidae | Te |  | Out_Su_Octo |  | *Octodon degus* |
| Rodentia | Petromuridae | Te |  | Out_Su_Hete |  | *Petromus typicus* |
| Rodentia | Thryonomyidae | Te |  | Out_Su_Hete |  | *Thryonomys gregorianus* |
| Rodentia | Thryonomyidae | Te |  | Out_Su_Hete |  | *Thryonomys swinderianus* |
| Scandentia | Tupaiidae | Te |  | Else |  | *Tupaia javanica* |

Table S5.

Newly sampled specimens (data added to the datasets of Amson *(36)* and Amson & Bibi *(10)*)

| Specimen number | Taxon | Common name | CT-scanner |
| --- | --- | --- | --- |
| SMNS-P-43253 | *Tegulariscaptor minor* | - | Bruker SkyScan1272 |
| SMNS-Z-MAM-160 | *Desmana moschata* | Russian desman | Bruker SkyScan1272 |
| SMNS-Z-MAM-43582* | *Crocidura russula* | Greater white-toothed shrew | Bruker SkyScan1272 |
| SMNS-Z-MAM-43552* | *Crocidura russula* | Greater white-toothed shrew | Bruker SkyScan1272 |
| SMNS-Z-MAM-49066* | *Suncus etruscus* | Etruscan shrew | Bruker SkyScan1272 |
| SMNS-Z-MAM-49068* | *Suncus etruscus* | Etruscan shrew | Bruker SkyScan1272 |
| UMUT104497* | *Scapanus orarius* | Coast mole | Shimadzu corp., inspeXio SMX-90CT |
| NHMUK_ZE_1958.3.11.5 | *Condylura cristata* | Star-nosed mole | Nikon XTH 225 ST |
| MNHN_1962-2031 | *Galemys pyrenaicus* | Pyrenean desman | Bruker SkyScan1272 |
| PIMUZ A/V 6312 | *Parascalops breweri* | Hairy-tailed Mole | Bruker SkyScan1272 |
| PIMUZ A/V 6313 | *Urotrichus talpoides* | Japanese shrew mole | Bruker SkyScan1272 |
| KIZ: AL1305058 | *Uropsilus gracilis* | Gracile shrew-like mole | Bruker SkyScan1272 |
| KIZ: H-110326013 | *Scaptonyx fusicaudus* | Long-tailed mole | Bruker SkyScan1272 |
| KIZ: LHS11041 | *Scapanulus oweni* | Gansu mole | Bruker SkyScan1272 |
| KIZ: AL1311181 | *Parascalops breweri* | Hairy-tailed mole | Bruker SkyScan1272 |
| KIZ: AL1311181 | *Parascaptor leucura* | White-tailed mole | Bruker SkyScan1272 |

Abbreviations: KIZ, Kunming Institute of Zoology, Chinese Academy of Sciences, Kunming, Yunnan, China; MNHN, Muséum national d’Histoire naturelle, Paris, France; NHMUK, Natural History Museum, London, UK; PIMUZ, Palaeontological Institute and Museum, Univ. of Zurich, Switzerland; SMNS, Staatliches Museum für Naturkunde Stuttgart, Germany; UMUT, The University Museum, the University of Tokyo.

*, only examined qualitatively.

Table S6.

Correlation between the investigated bone microanatomy parameters and body mass. Generalized least squares linear models outputs, using a model adjusted with the optimized Pagel’s lambda. Here the Tree 1A is used; other possible trees yielded similar results (see Material and Methods main text).

| **Humeral diaphysis mean humeral compactness** | | | | |
| --- | --- | --- | --- | --- |
|  | Value | Std.Error | t-value | p-value |
| Tree 1A |  |  |  |  |
| (Intercept) | 52.8006 | 5.329468 | 9.907292 | <0.0001 |
| Log Body Mass | 2.0936 | 0.964687 | 2.170243 | 0.0315 |
| **Humeral head mean trabecular thickness (mean Tb.Th)** | | | | |
| Tree 1A |  |  |  |  |
| (Intercept) | -1.3982347 | 0.09554025 | -14.635032 | <0.0001 |
| Log Body Mass | 0.2014476 | 0.0220499 | 9.135986 | 0 |
| **Humeral head bone volume fraction (BV/TV)** | | | | |
| Tree 1A |  |  |  |  |
| (Intercept) | 0.27136535 | 0.09626819 | 2.818847 | 0.0059 |
| Log Body Mass | 0.04744175 | 0.01643155 | 2.887235 | 0.0048 |
| **Humeral capitulum bone volume fraction (BV/TV)** | | | | |
| Tree 1A |  |  |  |  |
| (Intercept) | -0.07069168 | 0.25854771 | -0.2734183 | 0.7883 |
| Log Body Mass | 0.25763101 | 0.08751943 | 2.9437007 | 0.0101 |

Movie S1. (separate file)

CT-scan stack of the radius mid-diaphysis of a European mole (*Talpa europaea* SMNS-Z-MAM-32952). Roughly 70% of the cortical area at mid-diaphysis is made of compacted coarse cancellous bone (CCCB), appearing here as a disorganized inner region around the medullary cavity.

Data S1. (separate file)

Raw bone microanatomy measurements for the four investigated compartments: 1, humeral head; 2, humeral capitulum; 3, humeral diaphysis; 4, mid-lumbar centrum. See Table S4 for lifestyle and clade abbreviations.
